# Microbiota as diagnostic biomarkers: advancing early cancer detection and personalized therapeutic approaches through microbiome profiling

**DOI:** 10.3389/fimmu.2025.1559480

**Published:** 2025-05-08

**Authors:** Majid Eslami, Ramtin Naderian, Aisa Bahar, Ali Babaeizad, Solaleh Rezanavaz Gheshlagh, Valentyn Oksenych, Hamed Tahmasebi

**Affiliations:** ^1^ Cancer Research Center, Semnan University of Medical Sciences, Semnan, Iran; ^2^ Clinical Research Development Unit, Kowsar Educational, Research and Therapeutic Hospital, Semnan University of Medical Sciences, Semnan, Iran; ^3^ Department of Biochemistry, School of Medicine, Iran University of Medical Sciences, Tehran, Iran; ^4^ Student Research Committee, Semnan University of Medical Sciences, Semnan, Iran; ^5^ Faculty of Medicine, University of Bergen, Bergen, Norway; ^6^ School of Medicine, Shahroud University of Medical Sciences, Shahroud, Iran

**Keywords:** biomarkers, cancer, microbiota, microbiome profiling, personalized medicine

## Abstract

The important function of microbiota as therapeutic modulators and diagnostic biomarkers in cancer has been shown by recent developments in microbiome research. The intricate interplay between the gut microbiota and the development of cancer, especially in colorectal and breast cancers, emphasizes how microbial profiling may be used for precision treatment and early diagnosis. Important microbial signatures, including *Bacteroides fragilis* and *Fusobacterium nucleatum*, have been linked to the development and progression of cancer, providing important information on the processes behind carcinogenesis. Additionally, the influence of microbiota on the effectiveness of treatments such as immunotherapy and chemotherapy highlights its dual function in improving treatment outcomes and reducing side effects. To optimize treatment results, strategies including dietary changes and fecal microbiota transplantation (FMT) are being investigated. Despite these developments, there are still issues, such as individual variations in microbial composition, a lack of standardized procedures, and the requirement for reliable biomarkers. Integrating microbiome-based diagnostics with conventional approaches, such as liquid biopsies and machine learning algorithms, could revolutionize cancer detection and management. This review provides an overview of the current understanding of the host–microbe immunological axis and discusses emerging therapeutic strategies centered on microbiota modulation to support human health. Further research is essential to overcome existing challenges and fully realize the promise of microbiota-driven innovations in oncology.

## 1 Current research on microbiota in early cancer detection

Complex mechanisms that rely on the host’s tolerance are essential for preserving the symbiosis between the host and microbes in the intestine, which can involve the physical barriers of the gut tissues or the release of antimicrobial peptides and antibodies (1). Nevertheless, this interdependent relationship relies on a delicate and ever-changing balance, and disruptions in the communication between the body and the gut bacteria have been linked to various long-lasting disease like cancer (2).

The relevance and novelty of this review article its in-depth analysis of microbiota as both diagnostic and therapeutic biomarkers in oncology, with a specific focus on colorectal cancer (CRC), breast cancer, lung cancer, pancreatic cancer and prostate cancer. The manuscript integrates recent advances in microbiome research, it highlights how microbial signatures could transform early cancer detection and personalized treatment strategies (3). In terms of relevance, the study emphasizes the critical need for non-invasive, accurate, and tailored diagnostic methods in cancer care. It leverages the growing body of evidence linking microbiota composition to carcinogenesis, treatment outcomes, and disease prognosis. By integrating microbial biomarkers with conventional diagnostic methodologies such as liquid biopsies and machine learning algorithms, the paper offers a forward-looking perspective for addressing diagnostic and therapeutic challenges in oncology. Regarding novelty, the manuscript provides valuable insights into the dual role of microbiota in both tumor development and therapeutic outcomes. It not only revisits well-known microbial links, such as the association of *Fusobacterium nucleatum* with CRC, but also examines underexplored areas like the influence of microbiota on immunotherapy and chemotherapy responses (4). Furthermore, it explores innovative concepts, such as fecal microbiota transplantation (FMT) as a strategy to boost treatment efficacy, positioning it as a breakthrough avenue in precision oncology. The discussion surrounding the integration of microbiome profiling with AI-based diagnostic technology further strengthens the manuscript’s innovative contribution (5).

### 1.1 Contribution of the microbiota to cancer

Evidence is mounting to support the idea that the gut microbiome might affect the beginning and progression of tumor development. The gut microbiome is likely to impact the risk of cancer in multiple ways, including its involvement in cancer onset, advancement, dissemination, and reaction to treatments, as disturbances in host-microbiome equilibrium have been connected to several well-known cancer characteristics (1). Overall, these defining features consist of irregularities in the host’s metabolism and immune response, disturbances in the body’s equilibrium processes that result in persistent inflammation, and support for genetic instability and alterations (6). Building on this intricate relationship, the following section delves into how microbial exposure during early life shapes immune development and sustains homeostasis.

While a balanced microbiota contributes to immune homeostasis, its disruption—termed dysbiosis—has been increasingly recognized as a hallmark of several diseases. The microbiota significantly influences the development, progression, and treatment response across various malignancies, including breast, lung, pancreatic, and prostate cancers. The intricate relationship between microbial communities and oncogenic processes highlights the potential of microbiome profiling as a transformative tool in cancer diagnostics and therapeutics. In breast cancer, microbiota is recognized for its role at both local tissue and systemic levels in tumorigenesis. Research suggests that the breast microbiome modulates immune responses and metabolic processes (7). Beneficial bacterial strains, such as *Lactobacillus* and *Bifidobacterium*, are linked to anti-inflammatory effects that may provide protection against malignancy. Conversely, harmful species like *Escherichia coli* and *Staphylococcus aureus* can drive chronic inflammation and genomic instability (8).

The gut microbiota further influences breast cancer risk by regulating estrogen metabolism through the estrobolome, which manages systemic estrogen levels. Disruptions in the gut microbiome may elevate estrogen levels, promoting tumor growth. As a result, interventions targeting the microbiota such as probiotics, dietary modifications, and FMT are being explored as complementary strategies to enhance treatment success and minimize adverse effects (9). The research into xanthohumol anticancer properties complements the ongoing exploration of how dietary elements impact cancer progression and treatment via microbiome modulation. Similar to microbiome-targeted strategies like probiotics and dietary adjustments that enhance therapeutic outcomes, xanthohumol bioactive potential may play a role in anticancer effects by influencing gut microbiota and tumor microenvironments. This underscores the potential for a more personalized approach in oncology care (10). The research on hydrolyzed protein formulas and their impact on microbiota changes highlights the broader conversation about how dietary adjustments shape gut microbiome composition and contribute to overall health. Similar to how microbiome-focused nutritional approaches can boost the effectiveness of cancer therapies and strengthen immune functions, promoting microbial balance through personalized diets like hydrolyzed protein formulas underscores the promising role of microbiome-centered strategies in disease management and personalized treatment (11).

Lung cancer has also been linked to microbiota alterations, particularly through the gut-lung axis. The lung microbiome composition varies significantly between healthy individuals and those with lung cancer, with elevated levels of *Streptococcus* and *Veillonella* correlating with poor prognosis. Gut microbiota dysbiosis impacts immune surveillance by influencing inflammatory pathways and altering the production of short-chain fatty acids (SCFAs), crucial regulators of immune function (12). The effectiveness of immune checkpoint inhibitors (ICIs), such as anti-PD-1/PD-L1 therapies, has been shown to depend on gut microbiota composition. Certain bacteria, like *Akkermansia muciniphila* and *Bifidobacterium*, are associated with better immunotherapy responses, while microbial imbalances contribute to resistance. Consequently, microbiome profiling has emerged as a potential biomarker to predict which patients may benefit most from immunotherapy (13).

**Figure 1:**
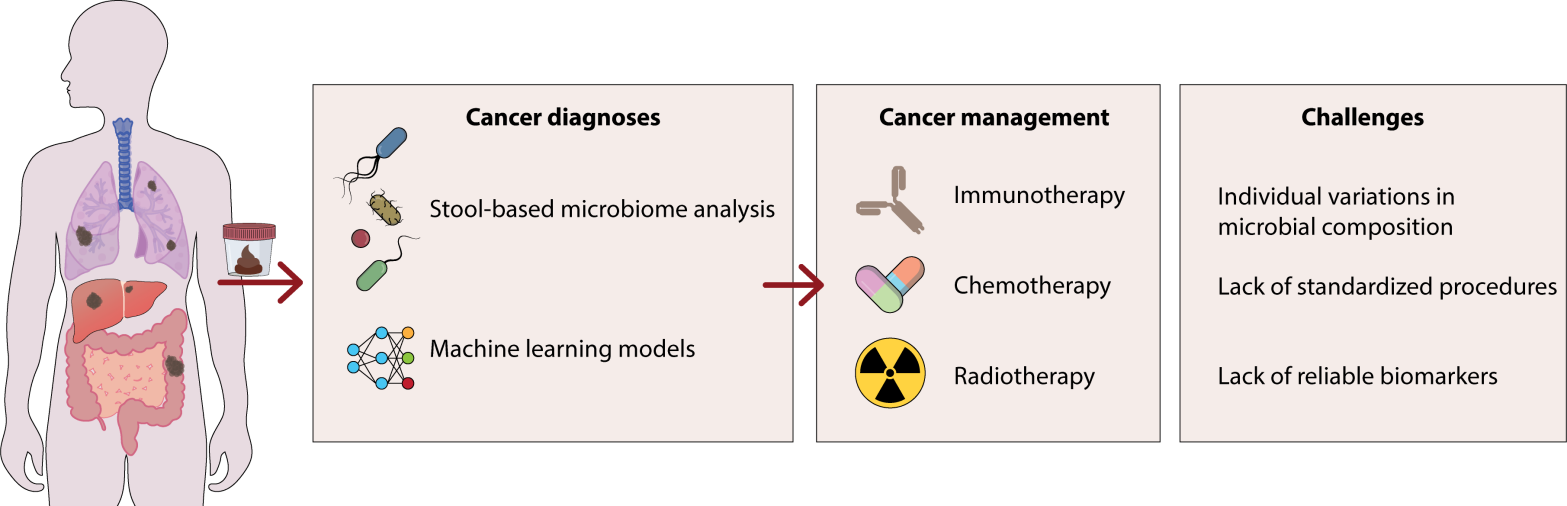
This figure shows the role of certain bacteria in the development of gastrointestinal cancer. These bacteria enhance the production of inflammatory cytokines including IL-6, iNOS and TGF-β through the activation of β-catenin/WNT signaling, which can lead to the development of colon cancers. Additionally, by producing DNA-damaging substances, these bacteria can lead to carcinogenic damage to the host DNA.

Research conducted with *in vitro* and murine models has indicated that *Fusobacterium nucleatum* may stimulate the proliferation of CRC cells and boost tumor growth rates. A number of explanations have been suggested to account for these findings. Through the interaction of FadA adhesin, *F. nucleatum* boosts cell division by binding to E-cadherin on the surface of CRC cells and initiates the oncogenic Wnt/β-catenin signaling cascade (3, 14). In addition to structural components of microbes, their metabolic byproducts—such as SCFAs and tryptophan derivatives—play pivotal roles in modulating host immune responses. SCFAs, such as butyrate, propionate, and acetate, are produced through the fermentation of dietary fibers and support the differentiation of regulatory T cells (Tregs) by promoting histone acetylation and increasing FOXP3 expression. Butyrate also strengthens the integrity of the intestinal epithelial barrier and reduces the production of pro-inflammatory cytokines, thereby supporting mucosal immune balance. Furthermore, tryptophan-derived indole compounds activate the aryl hydrocarbon receptor (AhR) on innate lymphoid cells (ILC3), leading to enhanced secretion of interleukin-22 (IL-22), which is vital for protecting mucosal immunity. Bile acid metabolites further influence immune cell signaling through receptors like the farnesoid X receptor (FXR) and TGR5, affecting dendritic cell function and the inflammatory response. Recent research has associated disturbances in these metabolite pathways with cancer progression, autoimmune disorders, and resistance to immune checkpoint inhibitors, underscoring their importance as diagnostic biomarkers and potential therapeutic targets. *F. nucleatum* promotes the growth of CRC by using Fap2 adhesin to attach to a specific sugar pattern and changing the behavior of immune cells in the tumor microenvironment through interaction with the TIGIT receptor. Activation of autophagy in CRC cells by *F. nucleatum* through LPS and Toll-like receptor 4 contributes to chemotherapy resistance (15). It is clear from the previously mentioned instances that variations in the gut microbiome and its potential association with CRC could offer a promising explanation for certain unexplained patterns in the disease’s occurrence. The causative relationship between bacterial traits in the gut microbiota and CRC is still unclear, and the evidence on the potential mechanisms involved is not strong enough to draw firm conclusions due to limitations (1, 16) (**Figure 1**).

For pancreatic cancer, particularly pancreatic ductal adenocarcinoma (PDAC), distinct microbiota signatures have been identified that impact disease progression and resistance to treatment. Salivary and gut microbiota analyses have identified increased levels of *Porphyromonas gingivalis* in pancreatic cancer patients suggest its use as a non-invasive biomarker for early detection. Chronic infections with *Helicobacter pylori* also appear to drive pancreatic carcinogenesis by sustaining inflammation and altering oncogenic pathways like Wnt/β-catenin signaling. In addition, the gut microbiota modulates chemotherapy response by affecting drug metabolism and immune system activity, highlighting the need for microbiome-based therapeutic innovations to improve treatment outcomes (17).

Prostate cancer progression has also been linked to microbiota dysbiosis, particularly regarding its effects on androgen metabolism. The gut microbiota plays a role in regulating systemic androgen levels, influencing tumor progression and the efficacy of androgen deprivation therapy (ADT) (18). Pro-inflammatory bacterial genera, such as *Bacteroides* and *Clostridium*, have been associated with disease progression, while protective effects have been linked to *Lactobacillus*. Microbial composition also affects the outcomes of hormonal therapy and radiotherapy, making it a promising target for therapeutic optimization. In summary, microbiota plays a crucial role across multiple cancer types by modulating oncogenic pathways, immune responses, and treatment efficacy. The growing body of microbiome research opens doors for early cancer detection, personalized therapy, and innovative treatment modalities. Future research should focus on integrating microbiome profiling into traditional diagnostic techniques, standardizing methodologies, and exploring the mechanistic pathways through which microbiota influence cancer progression and treatment outcomes. By establishing a deeper understanding of microbiota-cancer interactions, precision oncology can advance toward more individualized and effective cancer management strategies (19). Manipulating the microbiota presents substantial therapeutic opportunities, especially for boosting the effectiveness of cancer immunotherapy and chemotherapy. Strategies such as fecal microbiota transplantation (FMT), tailored probiotics, and diet alteration have demonstrated potential benefits. For instance, transferring microbiota from immunotherapy responders to non-responders has enhanced the effectiveness of checkpoint inhibitors in mouse models. Furthermore, particular probiotics, including *Akkermansia muciniphila* and *Bifidobacterium longum*, have been linked to better clinical results in patients undergoing PD-1 blockade treatment. Given these immunomodulatory effects, therapeutic modulation of the microbiota—through probiotics, prebiotics, or FMT—has gained considerable attention in clinical research.

## 2 Microbiota and cancer screening

### 2.1 Colorectal cancer screening and gut microbiome

CRC remains a leading cause of cancer-related deaths globally, with early detection playing a critical role in improving patient outcomes. Conventional screening methods such as colonoscopy, fecal occult blood tests (FOBT), and fecal immunochemical tests (FIT) are widely used to identify precancerous lesions and tumors. Despite their importance, these approaches come with notable drawbacks, including invasiveness, patient discomfort, and variability in sensitivity. Recent advancements in microbiome research suggest that analyzing gut microbial profiles could offer a complementary, less invasive tool for CRC screening (20).

The gut microbiome has emerged as a key player in the initiation and progression of CRC. Dysbiosis, or an imbalance in the microbial composition of the gut, has been associated with chronic inflammation, DNA damage, and immune system dysfunction factors that contribute to colorectal tumor development (21). Several bacterial species have been identified as potential biomarkers for CRC. *Fusobacterium nucleatum*, for example, fosters tumor growth by activating β-catenin signaling, modulating the immune response, and increasing resistance to chemotherapy. *Bacteroides fragilis* produces enterotoxins that can induce DNA damage and inflammation, elevating CRC risk. *Escherichia coli* (pks+ strain) harbors the pks pathogenicity island, responsible for producing colibactin, a compound linked to genomic instability. *Peptostreptococcus anaerobius* contributes to tumor progression by altering the tumor microenvironment, promoting cell proliferation, and triggering inflammation. These microbial markers not only provide insight into CRC development but also hold promise for enhancing early detection strategies (22).

Given the strong link between gut microbes and CRC, several microbiome-based diagnostic tools are under investigation. One promising approach is stool microbiome analysis a non-invasive method that identifies microbial signatures associated with CRC and demonstrates greater sensitivity and specificity than traditional fecal blood tests. This technique can detect key bacterial markers like *Fusobacterium nucleatum* and *Bacteroides fragilis* with precision. Another innovative option is liquid biopsy coupled with circulating microbial DNA (cmDNA) analysis, which involves detecting microbial DNA fragments in blood samples. This method allows for real-time monitoring of CRC progression and treatment efficacy while potentially being combined with circulating tumor DNA (ctDNA) analysis to enhance diagnostic accuracy. Additionally, the integration of machine learning with microbiome-based predictive models is revolutionizing CRC diagnostics (23).

### 2.2 Breast cancer screening and microbiota

Traditional breast cancer screening methods, including mammography, ultrasound, and MRI, dominate current practice. However, growing research highlights the potential of microbiota signatures as reliable biomarkers for early detection and risk categorization. Distinct microbial profiles in breast tissue, nipple aspirate fluid, and systemic circulation have been shown to differentiate between healthy individuals and those with breast cancer (24). Patients with breast cancer often exhibit microbial imbalances, marked by reduced levels of beneficial bacteria like *Lactobacillus* and *Bifidobacterium*, alongside an overrepresentation of pro-inflammatory and potentially pathogenic bacteria such as *Escherichia coli*, *Staphylococcus aureus*, and *Fusobacterium nucleatum*. These microbial disruptions may drive carcinogenesis by development chronic inflammation, oxidative stress, and immune system evasion (25). Additionally, the gut microbiota influences estrogen metabolism through the estrobolome, which can elevate systemic estrogen levels and heighten the risk of hormone receptor-positive breast cancer. Circulating microbial DNA (cmDNA) is emerging as a promising biomarker for non-invasive breast cancer detection. Metagenomic analyses of breast tissue and nipple aspirate fluid have identified unique microbial patterns that could signal malignancy at its earliest stages. Also, computational algorithms that combine microbiome profiles with clinical data are being refined to enhance diagnostic accuracy. There is also growing interest in microbiome-focused preventive strategies, such as probiotics and diet modifications, to reduce breast cancer risk (26).

### 2.3 Lung cancer screening and the respiratory microbiome

Low-dose computed tomography (LDCT) remains the cornerstone of lung cancer screening, but advancements in microbiome research suggest a complementary avenue for improved diagnostic precision. Alterations in the lung microbiome, heavily influenced by the gut-lung axis, are evident in lung cancer patients. Elevated occurrences of microbial genera such as *Streptococcus*, *Veillonella*, and *Prevotella* have been detected in the sputum and bronchoalveolar lavage fluid of affected individuals. These shifts offer distinct microbial markers for early disease identification. Gut microbiota imbalances also play a role in modulating systemic immune responses and the tumor microenvironment, impacting lung cancer progression. Liquid biopsy techniques that analyze microbial DNA from blood or respiratory secretions are emerging as non-invasive options to optimize screening efforts (25).

### 2.4 Pancreatic cancer screening and the oral-gut microbiome axis

The early detection of pancreatic cancer remains a significant challenge, primarily due to the lack of highly specific biomarkers. However, disruptions in the oral-gut microbiome axis have emerged as a potential diagnostic way, showing strong associations with pancreatic ductal adenocarcinoma (PDAC). Elevated levels of microbial species such as *Porphyromonas gingivalis*, *Aggregatibacter actinomycetemcomitans*, and *Fusobacterium nucleatum* have been identified in saliva samples from pancreatic cancer patients (27). Additionally, fecal microbiota analyses have highlighted an altered *Bacteroides*-to-*Firmicutes* ratio in these individuals. These microbial patterns could play a transformative role in advancing non-invasive screening methods for pancreatic cancer. Recent research suggests that bacterial metabolites, including lipopolysaccharides (LPS) and short-chain fatty acids (SCFAs), may contribute to the carcinogenesis of the pancreas by promoting chronic inflammation and immune evasion. The oral-gut microbiome axis also influences the tumor microenvironment by modulating pathways such as nuclear factor-kappa B (NF-κB) and Toll-like receptor (TLR) signaling, both of which are key promoters of tumor progression in PDAC. Salivary microbiota profiling, integrated with next-generation sequencing (NGS) and metagenomic analysis, shows promise for improving pancreatic cancer screening accuracy in terms of both sensitivity and specificity (28).

### 2.5 Prostate cancer screening and gut microbiota

The gut microbiota has also been closely linked to the development and diagnosis of prostate cancer. Dysbiosis, characterized by elevated levels of bacteria such as *Bacteroides*, *Clostridium*, and *Escherichia coli*, has been consistently observed in prostate cancer patients, alongside a decline in beneficial microbes like *Lactobacillus* and *Faecalibacterium*. Evaluating stool and urine microbiomes could complement traditional screening tools, such as prostate-specific antigen (PSA) testing and multiparametric MRI, offering improved diagnostic precision and risk assessment capabilities (29). Emerging evidence suggests the role of gut microbiota in regulating androgen metabolism and systemic inflammation, two critical factors in prostate cancer pathophysiology. The gut microbiome influences androgen bioavailability by modulating steroid hormone metabolism, potentially driving the initiation and progression of prostate tumors. Moreover, microbial-derived metabolites such as secondary bile acids and trimethylamine-N-oxide (TMAO) have been implicated in inflammatory pathways that may fuel tumorigenesis. Advancements in machine learning and artificial intelligence (AI) have facilitated the development of predictive models integrating microbiome data with conventional clinical markers. These models aim to enhance risk stratification and diagnostic accuracy. Future research should focus on microbiome-based interventions, including prebiotics, probiotics, and FMT, as promising approaches to address gut dysbiosis and improve prostate cancer prevention and treatment effectiveness (30).

## 3 Advancements in microbiome profiling for cancer therapy

### 3.1 Chemotherapy

Various research projects have explored how systemic cancer treatment impacts the makeup of gut bacteria in various cancer forms. Different types of cancer, including those affecting the gastrointestinal system and other areas, were examined in these studies, along with various chemotherapy treatments and settings (3). Systemic chemotherapy led to a rise in *Faecalibacterium prausnitzii* levels in midgut NET patients, as noted in those with neuroendocrine tumors. Even though this study utilized FISH to target specific species, recent articles employ sequencing-based methods to thoroughly analyze bacterial species composition (31).

The intestinal microbiota composition underwent a notable transformation following a five-day high-dose chemotherapy regimen for bone marrow transplantation, as revealed by sequencing the 16S rRNA gene, according to studies (14). A notable decrease was noted in the number of different microbe’s present, the estimated variety of microbes, and the diversity of microbes, suggesting a decrease in α-diversity as a result of chemotherapy. Therefore, it can be inferred that intensive chemotherapy led to a significant reduction in the variety of microorganisms and altered the composition of the microbial population (32). *Bacteroidetes* and *Proteobacteria* showed higher numbers at the phylum level, whereas *Firmicutes* and *Actinobacteria* displayed lower levels. Post-chemotherapy samples exhibited significantly higher levels of *Bacteroides* and *Escherichia* on the genus level when compared to pre-chemotherapy samples (33).

Additionally, there was a notable change from Gram-positive to Gram-negative bacteria observed in patients undergoing chemotherapy. It is worth noting that this research also mentioned that a few fewer common types of bacteria emerged following chemotherapy. Following chemotherapy, there was a reduction in *Blautia*, *Faecalibacterium*, *Roseburia*, and *Bifidobacterium*, which are known to promote health and have anti-inflammatory properties (3, 34). During induction chemotherapy, AML patients experienced significant alterations in their intestinal microbiota composition, as noted by Galloway-Peña et al. Through the analysis of the 16S rRNA gene, a notable decline in the diversity of microorganisms was detected, along with a reduction in the presence of the anaerobic bacteria *Blautia*. Conversely, chemotherapy led to a rise in the levels of *Lactobacillus* (35). One intriguing result of chemotherapy was the heightened occurrence of intestinal domination, where over 30% of intestinal bacteria are associated with a particular taxon. Opportunistic pathogenic bacteria, which are recognized for causing bacteremia, were responsible for 50% of the domination occurrences post-chemotherapy (32, 36). Moreover, a rise in opportunistic pathogenic genera was linked to elevated temporal variability within individual patients. Therefore, it was determined that the therapy caused a change towards a microbial composition closely resembling that of the gut microbiome in individuals without health issues. Youssef et al. (2018) obtained stool samples from individuals undergoing treatment for gastrointestinal tumors and healthy participants, contradicting previous studies that showed a decline in gut microbiota rather than an improvement. Tumors found in the stomach, small intestine, or rectum are classified as gastrointestinal neoplasms (37).

### 3.2 Immunotherapy

Certain research papers in human clinical trials were discovered that discussed shifts in the human gut microbiome while undergoing immunotherapy and using longitudinal sampling. Treatment with anti-PD-1, anti-CTLA-4, or interferon alpha-2b was given to patients diagnosed with metastatic or unresectable melanoma, renal cell carcinoma (RCC), non-small cell lung cancer (NSCLC), or neuroendocrine tumors (NET) (2, 16).

Moreover, there were no alterations in Shannon and Simpson α-diversity indices while undergoing ipilimumab therapy, indicating that the gut microbiota remained unaffected by the treatment. On the other hand, it is essential to point out that the quantity of fecal samples studied dropped to four over time. Although ipilimumab did not have a direct impact on the gut microbiota in this research, alterations in the gut microbiota were noted when colitis developed while on ipilimumab treatment. Hence, stool samples from seven colitis patients were obtained and juxtaposed with their original samples. Notable discrepancies in microbiota structure were detected among different family and genus categories (16).

The occurrence of ipilimumab-induced colitis in metastatic melanoma patients was linked to a notable decrease in the relative abundance of seven key genera (*Lachnospiracea incertae sedis*, *Ruminococcus, Clostridium* IV*, Blautia, Pseudoflavonifracto, Eubacterium*, unclassified and *Lachnospiraceae*). Moreover, a significant reduction in other bacteria, predominantly *Firmicutes*, was observed. A correlation was found between the microbiome in the human gut and the occurrence of colitis from systemic cancer therapy, which was linked to reduced α-diversity. Nevertheless, the disruptions in the microbes were probably due to the colitis rather than the treatment (6, 16).

**Figure 2:**
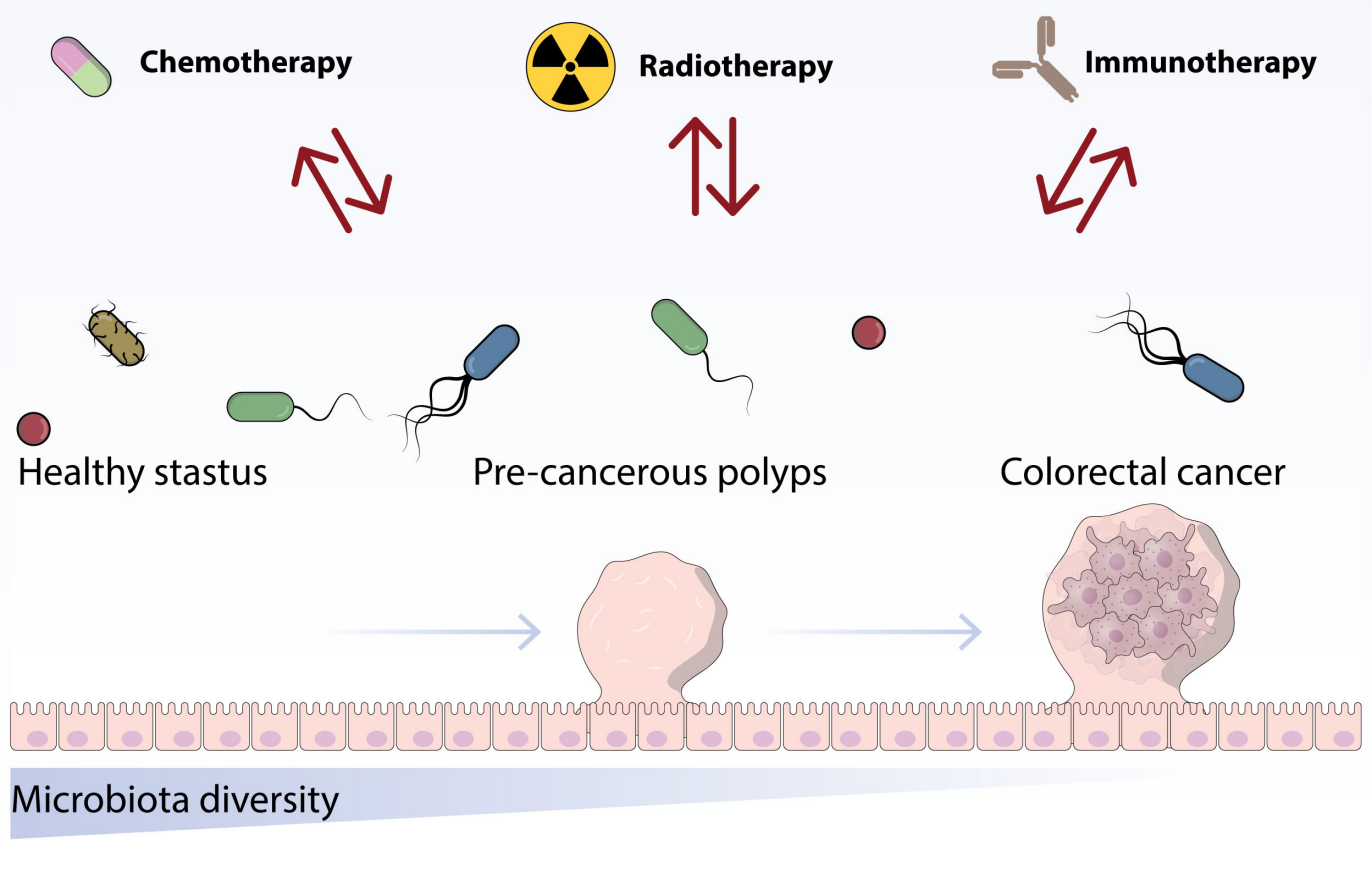
This figure indicates the role of chemotherapy and immunotherapy treatments on the structure and proportion of the established microbiota.

Surprisingly, studies have demonstrated that mouse tumors treated with ipilimumab exhibit improved responses to FMT from cluster C individuals rather than cluster B individuals. It is suggested that ipilimumab could potentially transform the enterotype to the more advantageous cluster C. In addition to the research conducted by Routy, Chaput, and Vetizou, three studies carried out extended monitoring of stool samples from a small group of melanoma patients undergoing immunotherapy (16) (**Figure 2**).

### 3.3 Hormonal therapy

There are other research projects that have looked into alterations in the human gut microbiome caused by hormonal treatment. Fecal samples were obtained from patients with neuroendocrine tumors who were undergoing treatment with somatostatin analogs, as documented by Puhalla et al. The abundance of certain bacterial groups in these subjects remained unchanged despite the administration of somatostatin analogs (38).

In a study by Sfanos et al. (2018), the intestinal microbiota of prostate cancer patients receiving androgen axis-targeted treatments was compared to those who were not on hormonal medication. In the realm of androgen axis-targeted therapies, treatment methods like gonadotropin-releasing hormone (GNRH) or therapies targeting the androgen receptor axis were utilized. Healthy controls, benign tumor patients, and untreated prostate cancer patients were part of the group not taking hormonal medication. Prostate cancer patients, whether on hormonal medication or not, exhibited similar α-diversity levels according to this study. Compared to the GNRH group and the group without hormonal medication, the ATT group displayed the lowest β-diversity (39). The findings suggest that the gut microbiota of patients on ATT was more alike compared to those not taking any medication. Moreover, ATT was linked to a reduction in β-diversity. There were notable changes in the types and amounts of bacteria in the stool samples of men who were on oral ATT in comparison to those not using any medication. Microbiome’s role in cancer therapy shown in **Table 1**.

**Table 1:** Microbiome’s role in cancer therapy.

Therapy Type	Microbiome Influence	Example Microbes	Potential Applications
Chemotherapy	Affects drug metabolism and resistance	*Fusobacterium nucleatum, Enterococcus hirae*	Enhancing drug response, reducing toxicity
Immunotherapy	Modulates immune checkpoint responses	*Akkermansia muciniphila, Bifidobacterium*	Predicting response to immune checkpoint inhibitors (ICIs)
Radiotherapy	Alters inflammation and tissue repair	*Faecalibacterium prausnitzii, Bacteroidetes*	Reducing radiation-induced toxicity
Hormonal Therapy	Impacts androgen and estrogen metabolism	*Lactobacillus, Clostridium*	Enhancing hormone therapy efficacy
FMT	Restores beneficial microbiota	Diverse gut microbiota	Improving immunotherapy and chemotherapy outcomes

## 4 Linking microbiota to cancer detection and progression

### 4.1 The role of the microbiome in pancreatic ductal adenocarcinoma pathogenesis

Considering the established connection between infectious agents, chronic inflammation, and cancer, it is not far-fetched to think of the microbiome as a factor in PDAC pathogenesis. Additionally, the absence of such microbes in a normal pancreas suggests that this idea could be valid (40, 41). A significant portion of cancer diagnoses, exceeding 16%, can be attributed to pathogen infections, with detailed descriptions available for most of the mechanisms. Eleven different types of “oncomicrobes” have been identified as causing cancer. Nevertheless, certain microorganisms implicated in the development of different types of cancer, like through the disruption of the Wnt/β-catenin signaling pathway, were similarly identified in PDAC, such as *Clostridium, Bacteroides*, and *E. coli*. Alterations in the Wnt/β-catenin signaling pathway are prominent in PDAC, presenting advantageous prospects for studying the microbiome’s involvement in PDAC tumor formation (40).

Beyond the triggering of oncogenic signaling, there are other viable pathways by which microbes could be involved in the development and maintenance of PDAC. Mutagenesis can lead to direct cancer-causing effects. Similarly, to oncogenic signaling, there is a parallel that supports the idea that microbes could have a hand in PDAC (40). The speculation is that *F. nucleatum* contributes to the carcinogenic and oncogenic processes of oral squamous cell carcinoma cells by activating the Ku70/p53 pathway and causing DNA double strand breaks. Indirectly, chronic inflammation could contribute to carcinogenic effects. Research has indicated a link between the development of prostate cancer and the connection between the oral microbiome, and periodontitis (40). According to another investigation, lipopolysaccharide (LPS), which is also known as microbe-associated molecular patterns, plays a role in promoting PC. In colonic cancer, microbial metabolites like short-chain fatty acids (SCFA) have been proven to transmit indirect oncogenic effects. It is crucial to reconsider the interaction between the microbiome and the immune system in terms of reshaping the immunogenic TME. Microbial infiltration has been identified in research as a possible cause of cancer development through the alteration of the immune system (40–42).

### 4.2 The impact of microbiome on cancer diagnostics

Detecting cancer at an early stage has been proven to reduce the chances of negative results and increase the chances of successful therapy. Different levels of invasiveness are seen in the current techniques used for cancer diagnosis, and a significant number of diagnoses continue to depend on invasive biopsies for verification. Research efforts have been directed towards identifying less intrusive approaches that can still ensure a high level of sensitivity (such as in cancer instances) and specificity (excluding non-cancer instances). Current research has emphasized the use of microbiomes for diagnosis, especially in cancer cases, where samples such as saliva, stool, and plasma can be collected more conveniently than other diagnostic approaches (15).

#### 4.2.1 Salivary microbiota

The analysis of the bacteria in saliva, known as salivary microbiota, is a highly studied diagnostic technique that can be conducted using noninvasive samples of saliva. Cancers in various parts of the body, such as the mouth, pancreas, and lungs, have been linked to the bacteria in saliva. The salivary microbiome of individuals with oral squamous cell carcinoma (OSCC) contains a unique mix of *Capnocytophaga gingivalis*, *Prevotella melaninogenica*, and *Streptococcus mitis*, which can be utilized as a diagnostic marker with an accuracy of 80% sensitivity and 83% specificity in contrast to healthy controls (43). Despite being considered commensal organisms, these three bacterial species were found to be notably increased in cancer patients. Recent research has suggested that *C. gingivalis* may have a role in promoting tumors, while the mechanisms for the other two species are not yet understood. The combination of certain oral bacteria, such as *Neisseria elongata* and *S. mitis*, can be used as a diagnostic tool for pancreatic cancer, distinguishing it from pancreatitis with good accuracy. Another study, more recent in nature, examined saliva samples from PDAC patients using advanced sequencing techniques, finding no significant variations in previously identified oral microbes like *P. gingivalis*, *N. elongate*, and *S. mitis*, raising doubts about their utility as diagnostic indicators (44).

One significant drawback of using the salivary microbiota for diagnosis is the uncertainty surrounding whether variations in microbes are a consequence or trigger of cancer. To clarify, these bacteria could exhibit a change in response to cancer only after its development, making them less reliable for detecting cancer in its early stages, although more research is necessary to unravel this link. Furthermore, the composition of the oral microbiome can vary significantly depending on the age, race/ethnicity, dietary habits, and lifestyle choices of individuals, emphasizing the importance of studying larger and more diverse patient cohorts to ensure precise diagnostic accuracy across all demographics (45).

#### 4.2.2 Fecal microbiota

The fecal microbiome, much like the oral microbiome, has been the subject of in-depth research and can be explored through noninvasive collection methods involving stool samples. Most studies focusing on using the fecal microbiome as a diagnostic indicator have primarily centered on CRC (46). The fecal microbiome, much like the oral microbiome, has been the subject of in-depth research and can be explored through noninvasive collection methods involving stool samples. The main focus of research on using the fecal microbiome as a diagnostic tool has been on CRC (3).

In addition to this, the fecal microbiome has been employed to examine biomarkers for different forms of cancer, albeit to a lesser extent. In the gut microbiome of lung cancer patients, a study using 16S rRNA analysis revealed a reduction in certain bacterial genera and an increase in 11 bacterial genera. Further investigation is necessary to uncover the rationale behind why these categories might act as gut markers for lung cancer and to validate this concept with larger groups of patients (45).

#### 4.2.3 Plasma cell-free DNA

A new trend has emerged in the field of cancer diagnosis, shifting the focus towards the utilization of cell-free DNA from human plasma, departing from the traditional methods used in microbially-rich environments. Liquid biopsies, or cell-free DNA tests, have experienced a rapid surge in application, transitioning from their initial role in prenatal examinations to broader health issue assessments (47).

Nevertheless, these examinations concentrate on the use of circulating tumor DNA found in the bloodstream. Recent studies have delved into the presence of microbial DNA sequences in plasma as a potential diagnostic tool, following earlier research that established a link between microbes and cancer. Microbial DNA was identified in the plasma of three early-onset breast cancer patients through next-generation sequencing, with the majority coming from bacteria, along with contributions from fungi and viruses (33, 45). The bacterial DNA detected in the blood of cancer patients differed significantly from that of the control group, showing higher levels of *Pseudomonas* spp. and *Sphingomonas* spp. in cancer patients, while *Acinetobacter* spp. were more prevalent in the controls. The authors suggest that more extensive studies with a larger sample size are necessary before any definitive conclusions can be made. Further investigations have revealed alterations in microbial patterns in the bloodstream of cancer patients without genetic mutations, with researchers noting the potential presence of microbial sequences in various blood components apart from plasma (48).

Even though there have been advancements in utilizing various microbiomes for cancer detection in experiments, it is probable that these microbiome-centered diagnostic strategies will be employed in conjunction with other established methods like imaging or biopsies, rather than as standalone diagnostic tools. Detecting cancer in its early stages and reducing the need for invasive tests could be significantly enhanced by these techniques. It is anticipated that diagnostic strategies centered on the microbiome could emerge as autonomous tools as the scientific community delves deeper into the intricate connections between microbiomes and cancer (48). Diagnostic methods involving microbiota shown in **Table 2**.

**Table 2:** Diagnostic methods involving microbiota.

Diagnostic Method	Sample Type	Cancer Type	Sensitivity & Specificity	Advantages	Limitations
Stool Microbiome Analysis	Stool	Colorectal, Pancreatic, Prostate	High for CRC (≥90%)	Non-invasive, easy collection	Affected by diet and lifestyle
Salivary Microbiome Profiling	Saliva	Pancreatic, Oral, Lung	Moderate	Easy to collect, low-cost	Microbiome varies over time
Blood Microbial DNA (cmDNA)	Plasma	Breast, Lung, Colorectal	Emerging	Minimally invasive	Low microbial DNA concentration
Bronchoalveolar Lavage (BAL) Microbiome	BAL Fluid	Lung	Moderate	Direct tumor site sampling	Invasive procedure required
Urinary Microbiome Analysis	Urine	Prostate	Emerging	Non-invasive	Requires further validation

## 5 Microbiota as biomarkers for early cancer diagnosis

Finding the perfect biomarkers for various diseases, such as certain forms of cancer, is seen as a challenging endeavor. Most current sampling techniques for cancer tissues do not have the capability to distinguish patients who will not respond to therapy and face challenges in accurately categorizing cancer subtypes because of the variability in tumors both within and among patients. It is important for a biomarker to be readily quantifiable, non-intrusive, and inexpensive (3, 44).

The human microbiome, specifically the gut microbiome, offers a non-intrusive method for pinpointing disease biomarkers that have the potential to detect various illnesses in their initial phases. Also, the utilization of microbiome-related biomarkers, in conjunction with clinical data and other biomarkers, can boost the accuracy of disease classification. To illustrate, some microbes are recognized for their contribution to the shift from adenoma to carcinoma in certain cancers, such as CRC. Microorganisms like these can serve as useful indicators for the effectiveness of treatments for CRC (3, 36, 47).

Besides microbiome-derived biomarkers, mast cells (MCs), microRNAs (miRNAs), imaging methods, and machine-learning models are also gaining attention as non-invasive biomarkers for disease diagnosis and prognosis, offering a glimpse into the future of precision medicine. Occasionally, the human microbiota interacts with different genetic or chemical markers. Changes in small RNA patterns found in feces of individuals with CRC are indicative of the types of bacteria present in their stool samples. Therefore, the utilization of several interlinked biomarkers in a network could enhance the efficiency of current biomarkers (46, 49). Infectious diseases were replaced by cancer and cardiovascular diseases as the leading causes of death on a global scale, due to an epidemiological shift. Currently, there are 19 million new cancer cases and 10 million fatalities globally, with an expected 50% surge in the next 20 years, especially in countries with low levels of human development (49, 50).

### 5.1 Colorectal cancer

The microbiome has emerged as an essential biomarker for cancer detection, prognosis, and treatment response across multiple types, including colorectal, breast, lung, pancreatic, and prostate cancers. In colorectal cancer, specific microbial signatures such as *Fusobacterium nucleatum*, *Bacteroides fragilis*, and *Escherichia coli* containing the pks pathogenicity island are linked to tumorigenesis through mechanisms like chronic inflammation, DNA damage, and immune modulation (51). Among them, *Fusobacterium nucleatum* is extensively studied for its role in activating the β-catenin signaling pathway and promoting immune evasion, making it a highly promising non-invasive biomarker detectable in stool and tissue samples. Recent advancements in microbiome-based stool tests, when combined with standard methods like the fecal immunochemical test (FIT), have notably improved diagnostic precision. Moreover, circulating microbial DNA (cmDNA) in plasma is being explored as a liquid biopsy biomarker for colorectal cancer. The microbiome also significantly influences treatment outcomes, with *Fusobacterium nucleatum* enrichment linked to resistance against fluoropyrimidine-based chemotherapy. In contrast, beneficial microbes such as *Akkermansia muciniphila* are associated with better responses to immune checkpoint inhibitors (52, 53).

### 5.2 Breast cancer

In breast cancer, the microbiome contributes to tumor development through both local and systemic mechanisms, particularly its impact on estrogen metabolism via the gut-associated estrobolome. Research findings indicate that breast cancer patients exhibit distinct microbial compositions, including reduced levels of anti-inflammatory species like *Lactobacillus* and *Bifidobacterium*, alongside elevated levels of pro-inflammatory and oncogenic bacteria such as *Escherichia coli*, *Staphylococcus aureus*, and *Fusobacterium nucleatum* (54). This disruption of gut microbial diversity alters estrogen metabolism, increasing systemic estrogen levels and elevating the risk of hormone receptor-positive breast cancer. Metagenomic analyses of breast tissue, nipple aspirate fluid, and blood samples have successfully identified microbial DNA that differentiates malignant from benign conditions, supporting their potential as biomarkers for early cancer detection. Additionally, gut microbiota composition appears to modulate the efficacy of endocrine therapies such as selective estrogen receptor modulators and aromatase inhibitors where higher microbial diversity correlates with more effective drug metabolism and treatment responses (55).

### 5.3 Lung cancer

In lung cancer, research into the gut-lung axis has highlighted its role in shaping immune responses and tumor dynamics. Patients with lung cancer exhibit pronounced alterations in both pulmonary and gut microbiota. For example, elevated levels of species such as *Streptococcus*, *Veillonella*, and *Prevotella* in bronchoalveolar lavage fluid and sputum have been proposed as potential diagnostic markers. Gut microbiota also influences the efficacy of immune checkpoint inhibitors like PD-1/PD-L1 therapies; an abundance of beneficial microbes such as *Akkermansia muciniphila* and *Bifidobacterium* enhances immunotherapy responses, whereas dysbiosis involving species like *Clostridium* and *Ruminococcus* correlates with resistance. Additionally, circulating microbial DNA in plasma is being investigated as a non-invasive diagnostic tool for lung cancer. Using machine learning algorithms to integrate microbiome data has further improved diagnostic accuracy (56).

### 5.4 Pancreatic cancer

In pancreatic cancer, the microbiome significantly influences disease development and progression. Microbial signatures from oral, gut, and pancreatic tumor microbiota show promise as diagnostic biomarkers. Increased levels of *Porphyromonas gingivalis*, *Aggregatibacter actinomycetemcomitans*, and *Fusobacterium nucleatum* in saliva samples are associated with pancreatic ductal adenocarcinoma (PDAC), pointing to an oral-gut microbiome axis involved in tumorigenesis (27). Gut dysbiosis characterized by a higher *Bacteroides*-to-*Firmicutes* ratio has also been linked to pancreatic cancer progression. Microbial metabolites such as lipopolysaccharides and short-chain fatty acids contribute to carcinogenesis by driving chronic inflammation and immune evasion. Importantly, certain bacteria like *Gammaproteobacteria* produce enzymes capable of inactivating gemcitabine, a primary chemotherapy drug for pancreatic cancer, thereby contributing to drug resistance. Strategies aimed at modifying the microbiota such as antibiotics or FMT are being explored to enhance chemotherapy effectiveness (57).

### 5.5 Prostate cancer

The gut microbiome has been increasingly implicated in the progression of prostate cancer through its effects on androgen metabolism and systemic inflammation. Individuals with prostate cancer exhibit distinct microbial alterations in stool and urine samples, characterized by an overabundance of *Bacteroides*, *Clostridium*, and *Escherichia coli* microbes linked to chronic inflammation and tumor growth alongside a reduction in beneficial species such as *Lactobacillus* and *Faecalibacterium*. Current research indicates that the composition of the gut microbiota can influence the effectiveness of androgen deprivation therapy (ADT), with certain bacteria capable of metabolizing androgens, potentially impacting treatment outcomes. Additionally, microbiome-targeted strategies, such as probiotics and dietary interventions, are being explored as promising therapeutic approaches to enhance prostate cancer management (58). Microbial biomarkers across cancer types shown in **Table 3**.

**Table 3:** Microbial biomarkers across cancer types.

Cancer Type	Key Microbial Biomarkers	Role in Carcinogenesis	Detection Method
Colorectal Cancer	*Fusobacterium nucleatum, Bacteroides fragilis, Escherichia coli (pks+)*	Promotes inflammation, immune evasion, and DNA damage	Stool microbiome analysis, liquid biopsy
Breast Cancer	*Lactobacillus, Bifidobacterium, Escherichia coli, Staphylococcus aureus*	Alters estrogen metabolism and immune modulation	Nipple aspirate fluid, blood microbiome
Lung Cancer	*Streptococcus, Veillonella, Prevotella*	Affects immune surveillance and inflammation	Bronchoalveolar lavage, sputum analysis
Pancreatic Cancer	*Porphyromonas gingivalis, Fusobacterium nucleatum*	Induces chronic inflammation and immune suppression	Salivary microbiome, stool analysis
Prostate Cancer	*Bacteroides, Clostridium, Lactobacillus*	Influences androgen metabolism and immune modulation	Urine microbiome, stool analysis

## 6 Personalized cancer treatment through microbiome insight

The human microbiome plays a pivotal role in shaping how individuals respond to cancer treatments, influencing drug metabolism, immune system activity, and tumor behavior. While traditional cancer therapies often adhere to standardized approaches, research increasingly highlights the microbiome’s capacity to significantly affect treatment outcomes. By integrating microbiome insights, researchers and clinicians aim to create personalized cancer therapies that not only improve effectiveness but also reduce side effects (59). Every person has a unique microbial ecosystem that interacts intricately with their immune system and metabolic processes, shaping their response to cancer treatments. Gut bacteria, for instance, affect the absorption and breakdown of chemotherapy drugs. Some microbes produce enzymes that degrade or deactivate these drugs, leading to treatment resistance. In CRC, *Fusobacterium nucleatum* has been identified as a contributor to chemoresistance, while *Akkermansia muciniphila* has shown promise in boosting chemotherapy effectiveness by enhancing immune activation. Similarly, the success of ICIs like PD-1 and CTLA-4 inhibitors is linked to specific gut bacteria. Patients with high levels of *Bifidobacterium* and *Faecalibacterium prausnitzii* often exhibit improved responses to immunotherapy, whereas those suffering from dysbiosis may experience diminished treatment benefits. In the case of radiotherapy, certain beneficial microbes such as species of *Lactobacillus* help reduce radiation-induced inflammation and maintain gut barrier integrity, mitigating toxicity during treatment (60).

Beyond observing the microbiome’s impact, researchers are now investigating ways to actively modify it to optimize cancer treatment outcomes. Emerging innovations include synthetic probiotics engineered to deliver therapeutic compounds, prebiotic-based solutions aimed at fostering beneficial microbial populations, and CRISPR-powered technologies to alter bacterial genes involved in drug metabolism. Live biotherapeutic products (LBPs) genetically modified microbes designed to enhance immune modulation and chemotherapy responses are also under development. These approaches signal a transformative shift toward incorporating microbiome engineering as a key component of precision oncology (25). Advancements in sequencing technologies, such as metagenomic and shotgun sequencing, now enable real-time tracking of a patient’s microbiome during treatment. This development allows clinicians to monitor microbial dynamics, predict potential drug resistance, and tailor personalized interventions as needed. Additionally, machine learning tools are being integrated with microbiome datasets to identify predictive biomarkers, categorize patients based on treatment responses, and fine-tune therapeutic planning. By identifying ARPC1A as a prognostic biomarker in low-grade glioma, reinforcing the move toward personalized cancer treatment. By integrating microbiome and genetic insights, clinicians can design therapies tailored to an individual’s unique tumor profile, moving away from the limitations of a one-size-fits-all approach (61).

However, the promise of microbiome-driven cancer therapy, several challenges remain. Microbiome diversity across individuals shaped by diet, genetics, antibiotics, and other factors complicates the creation of universally effective interventions. Furthermore, the absence of standardized protocols for microbiome sampling, sequencing methods, and data interpretation poses challenges for clinical implementation. Regulatory hurdles compound these issues, particularly for novel therapies like FMT and engineered probiotics, both of which require comprehensive validation in large-scale clinical trials before achieving broader approval in oncology settings (62). Utilizing the microbiota to enhance cancer treatments has emerged as a new approach in personalized medicine. While current results are promising, challenges remain including a limited understanding of how microbiota affects therapy, undefined microbial biomarkers, and no consensus on the best modulation methods. Moreover, most research focuses on bacteria, overlooking the roles of commensal viruses, fungi, and archaea in cancer. Modern strategies integrating microbiome in cancer treatment shown in **Table 4**.

**Table 4:** Modern strategies integrating microbiome in cancer treatment.

Cancer Type	Microbiome-Related Strategies
Colorectal Cancer	• Antibiotic targeting of *Fusobacterium nucleatum* • Prebiotics/Probiotics to enhance chemotherapy • FMT for immunotherapy improvement • Stool-based microbiota screening
Breast Cancer	• Estrobolome-targeting probiotics to optimize endocrine therapy • Gut microbiota modulation for reducing chemotherapy toxicity • Liquid biopsy for microbial DNA analysis
Lung Cancer	• *Akkermansia muciniphila*-driven immunotherapy enhancement • Selective antibiotic use to prevent ICI resistance • Microbial profiling in BAL fluid for early detection
Pancreatic Cancer	• Antibiotic targeting of *Gammaproteobacteria* to improve gemcitabine response • Salivary microbiota biomarkers for early diagnosis • FMT for gut microbiota restoration
Prostate Cancer	• Gut microbiome modulation for androgen deprivation therapy • Urinary microbiota analysis for non-invasive screening • High-fiber diets and probiotics for treatment support

## 7 Microbiota’s role in predictive oncology and custom therapies

The microbiota has emerged as a critical factor in oncology, influencing cancer development, progression, and treatment response. Its predictive role is being increasingly studied across several aspects of cancer care. Certain microbiota, particularly in the gut, have been linked to cancer development. For example, certain strains of *E. coli* possess a pathogenicity island known as *pks*, which contains genes responsible for producing the genotoxic compound colibactin. This metabolite could potentially be used as a biomarker to predict the risk of colon cancer. Approximately 16% of cancer types have been caused by microbial organisms, *Helicobacter pylori* is known to play a role in the development of adenocarcinoma in gastric and duodenal epithelial cells, as well as gastric cancer (63, 64). *Streptococcus*, *Haemophillus*, and *Bifidobacterium* have been identified as oral cancer biomarkers, using sequence-based techniques (63). However, the precise ways bacteria contribute to cancer development are still unclear, likely due to the complexity of bacterial communities and their interaction with living cells, so developing non-invasive tests to analyze microbiome composition could help predict cancer risk and prognosis in real-time (19, 63).

The composition of a patient’s microbiota can also predict their response to cancer treatment. Chemoresistance caused by *F. nucleatum* in mice may also be seen in human CRC patients. Higher levels of *F. nucleatum* in tumors are associated with quicker cancer recurrence and can predict CRC recurrence more accurately than the traditional cancer staging system (65, 66). Meanwhile, as the relationship between CDD-L, found in gram-negative bacteria, and gemcitabine chemoresistance is well known, a clinical study exhibited the potential role of intratumoral lipopolysaccharide, a cell wall component of Gram-negative bacteria, to be a negative predictor of gemcitabine efficacy in advanced pancreatic cancer (67). So, antibiotics targeting the CDD-L-producing bacteria improve gemcitabine response in patients with pancreatic ductal adenocarcinoma (PDAC) (65, 67). Preclinical studies indicated that prebiotics inulin and oligofructose increase the cytotoxic effects of 5-fluorouracil and CTX. Prebiotic consumption may enrich immune-effective bacteria. Clinical studies on the impact of prebiotics on chemotherapy are lacking So, Future investigations are essential to address the clinical safety of prebiotics (65).

Previous studies investigated the role of microbiota on the clinical response of ICIs and tried to elucidate the principle mechanism of dysbiosis of microbiota in the immune response. The microbiota that is effective in cancer immunotherapy response is not the same in clinical studies. This variation may be due to not having standard methods across studies. However, some of the bacteria identified from human studies, including *A. muciniphila*, *B. intestinihominis*, and *B. thetaiotaomicron*, were shown to improve therapeutic response through immunomodulation (65). As mentioned before *F. nucleatum* has been linked to poor prognosis in CRC, but it enhances the efficacy of immunotherapy. Thus, *F. nucleatum* is a potential bacterial biomarker for CRC and also immunotherapy response (63). Various therapeutic approaches have been tailored to modulate the microbiome to enhance the effectiveness of immunotherapy, based on recent discoveries in this field. FMT treatment is the transplantation of an individual’s gut microbiome usually from responders to non-responders. Its key benefit is that it can alter the microbiome, even without a clear understanding of the underlying mechanisms. Human and animal studies indicated that FMT can boost both immunotherapy response and irAEs, such as colitis (65, 68). Dietary modulation like high fiber diet in metastatic non-small cell lung carcinoma (NSCLC) patients receiving ICI, increased *Bifidobacterium* species which are another immunogenic bacteria associated with improved anticancer immunity (65). In addition, choosing specific antibiotic regimens can potentially have indirect anticancer benefits and help minimize complications during cancer therapies by targeting harmful microorganisms that either contribute to cancer development or lead to adverse events (69, 70). The research on immunoadjuvant-functionalized metal–organic frameworks (MOFs) complements the growing understanding of how microbiome insights can improve cancer immunotherapy. Similar to how beneficial gut microbes such as *Akkermansia muciniphila* and *Bifidobacterium* enhance the efficacy of immune checkpoint inhibitors, MOFs provide an innovative approach to modulating tumor immunity. This holds promise for advancing precision oncology and transcending conventional therapeutic methods (71).

The gut microbiome can be a double-edged sword for irAEs. Researches indicated that Checkpoint inhibitor colitis (CIC), the most frequently reported irAE, can be developed by *Faecalibacterium prausnitii* and *Bacteroidetes* or be protected by *B. fragilis* (68). In a clinical study, chaput et al. demonstrated that in metastatic melanoma patients receiving Ipilimumab, a higher abundance of *Faecalibacterium* and other *Firmicutes* was associated with better response with more colitis. Therefore, understanding the relationship between different microbes and side effects is particularly important for individualized palliation of these adverse events (69).

## 8 Challenges and future of microbiota in cancer diagnostics

Investigate the current challenges in using microbiota for cancer diagnostics, such as variability in microbial communities and standardization issues. Discuss potential future directions and solutions. Early diagnosis of cancer usually guarantees a better prognosis and increased treatment success rate. The available diagnosis techniques often involve invasive biopsies. This calls for research into less invasive techniques. Such techniques are those that have high sensitivity, which detects cancer patients, and high specificity, which rules out non-cancer patients. Recent studies have tested the ability of microbiomes to diagnose cancer through non-invasive means, using easily accessible samples such as saliva, stool, and plasma-a promising alternative to traditionally used invasive techniques. In fact, the use of microbiota for diagnostics to the cancer is mirrored by a remarkable promise combined with major challenges. Some limiting factors for broad use of microbiota-based diagnostics in cancer include variation in microbial communities, discrepancies in methods of approaches and lack of uniformity in approaches (72).

Challenges for microbiota-based diagnostics the composition of the microbiota varies between individuals and among populations and is highly modulated by diet, age, geographic location, medication use, and lifestyle (72, 73). These factors may mask any association between specific microbial signatures and cancer. Whereas detection of *Fusobacterium* enrichment in CRC lesions and stools has been reported in various populations, microbial community variation among individuals and populations complicates generalization. Such variability requires even finer control and adjustments in the design of studies to account for such variability (74).

Despite its potential, microbiome-driven cancer treatment faces significant challenges, mainly regarding standardization and reproducibility. One major issue is the natural variability of the microbiome across individuals, shaped by factors such as genetics, diet, geographic location, medication use (notably antibiotics), and underlying health conditions. This variability complicates efforts to create universally effective therapeutic approaches centered on the microbiome. A critical obstacle is the lack of standardized protocols for microbiome analysis. Variations in sampling techniques whether using stool, saliva, blood, or tumor tissue as well as differences in DNA extraction methods and sequencing technologies, contribute to inconsistent research outcomes. Additionally, the diverse range of bioinformatics tools employed to process microbiome data can lead to biases and conflicting interpretations. Without unified methodologies, it remains challenging to transition microbiome-focused discoveries from the research phase into practical clinical use (75).

Another key challenge is the identification of clinically relevant microbial biomarkers. While studies often report links between specific bacterial species and cancer treatment outcomes, these associations are not always causative. The intricate dynamics of host-microbiome interactions make it difficult to determine whether particular microbes actively affect treatment responses or simply reflect other underlying factors. Large-scale, and carefully controlled clinical trials are essential to validate these findings before microbial biomarkers can inform routine clinical practices. Regulatory challenges add another layer of complexity to adoption of microbiome-based interventions. FMT, for example, has shown encouraging results in restoring microbial balance and improving responses to immunotherapy. However, concerns about long-term safety and potential transmission of harmful pathogens remain. Similarly, engineered probiotics and drugs designed to modulate the microbiome require rigorous safety evaluations before they can receive regulatory approval for use in cancer care (76).

Standardization issues: To overcome these challenges, the field must move toward standardized microbiome research frameworks that facilitate reproducibility and enable clinical application. Establishing universal guidelines for microbiome sampling, sequencing, and data analysis will significantly enhance the comparability of findings across studies. Moreover, integrating machine learning and AI to analyze microbiome data may help identify microbial signatures that can predict treatment responses more accurately. Another critical area for future development is the creation of personalized microbiome-based therapies personalized to the unique needs of each patient. Innovations in microbiome engineering, such as developing synthetic probiotics and microbiota-specific drugs, hold promise for delivering more precise therapeutic options. Additionally, integrating microbiome analysis into multimodal cancer treatment plans by combining it with data from genomics, proteomics, and metabolomics can pave the way for a fully individualized approach to cancer care (77–79). Personalized cancer treatment strategies shown in **Table 5**.

**Table 5:** Personalized cancer treatment strategies.

Cancer Type	Microbiome-Based Strategy	Mechanism of Action	Expected Benefit
Colorectal Cancer	Antibiotics targeting *Fusobacterium nucleatum*	Reducing tumor-promoting bacteria	Enhancing chemotherapy response
Breast Cancer	Probiotics for estrobolome modulation	Regulating estrogen metabolism	Improving hormone therapy efficacy
Lung Cancer	*Akkermansia muciniphila* enhancement via diet	Boosting immune response	Enhancing ICI effectiveness
Pancreatic Cancer	Targeting *Gammaproteobacteria* to improve chemotherapy	Reducing drug inactivation	Enhancing gemcitabine response
Prostate Cancer	High-fiber diet and probiotics	Modulating androgen metabolism	Improving androgen deprivation therapy outcomes

## 9 Combining microbiome data with traditional cancer tests

Explain how integrating microbiome data with conventional diagnostic methods can improve cancer detection accuracy. Provide examples of combined approaches and their benefits. The human microbiome communities of bacteria, viruses, fungi, and other microorganisms residing in and on our bodies has been shown to be linked with several cancer types. For instance, particular microbial signatures, such as the overrepresentation of *F. nucleatum* in CRC, have been consistently observed in both tissue and stool samples (80).

Microbial cell-free DNA (cfDNA) from liquid biopsies is emerging as a minimally invasive approach for early cancer detection. Traditionally, cancer detection using liquid biopsies targets tumor-derived markers like circulating tumor DNA (ctDNA). However, ctDNA can be difficult to detect, especially in early-stage cancers due to low concentrations. Recent advances leverage non-human cfDNA from the microbiome, analyzed via whole-genome sequencing (WGS), to detect cancer. Microbial cfDNA, released by tumor-associated bacteria, viruses, and fungi, offers an additional layer of diagnostic potential, with studies demonstrating its ability to distinguish between cancer and non-cancer cases using machine learning models (81).

Although, the use of stool-based microbiome analysis in conjunction with traditional CRC screening methods has shown great promise. In a study that combined a fecal immunochemical test (FIT), which detects blood hidden in the stool, with microbiome analysis, researchers found a significant improvement in the accuracy of CRC diagnosis. The sensitivity of FIT alone for detecting CRC is approximately 79%, but when combined with microbiome markers such as *Fusobacterium* or *Porphyromonas*, the sensitivity improved to more than 90% (82).

Also, Machine learning (ML) models use feature vectors to make predictions, where the input vector consists of M features, and the model predicts a target value based on a decision function. The primary goal of ML models is to minimize the loss function, which measures the discrepancy between predicted and actual outcomes. These tasks are categorized into classification or regression, depending on whether the target variable is categorical or numerical. In cancer-related microbiome research, classification is the most common approach, focusing on cancer diagnosis or tumor type identification. Regression tasks, though less frequent, are used for outcomes such as predicting survival time or tumor growth in experimental models.

### 9.1 Support vector machines

Support vector machines are popular for cancer-related microbiome research, particularly for identifying biomarkers. They work by defining a decision boundary (hyperplane) and, using techniques like the “kernel trick,” can handle non-linear data (83). SVMs have shown good performance in CRC prediction but are less interpretable than other models like Random Forests (84).

### 9.2 Decision tree-based models

Decision tree-based models especially Random Forests, are widely used in cancer-microbiome studies. Random Forests reduce variability by averaging the predictions of multiple trees, providing better generalization than single Decision Tree (85). They have successfully identified various cancers, such as colorectal and lung cancer (85). Boosting further improves performance by building models sequentially and adjusting for errors, with models like Explainable Boosting Machines (EBMs) enhancing interpretability (86).

### 9.3 Logistic regression

**Figure 3:**
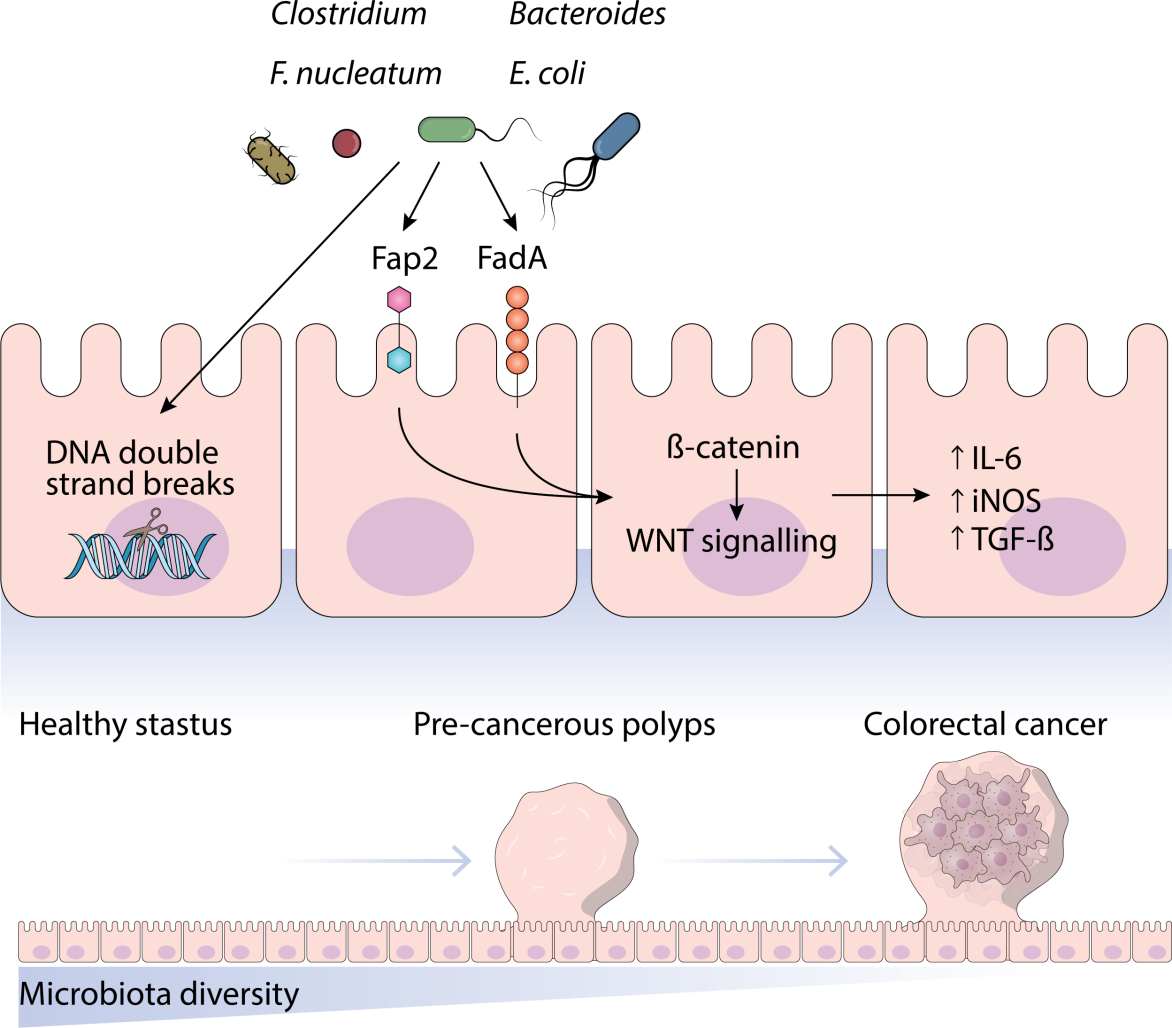
This figure highlights the role of microbiota in cancer diagnosis and treatment selection and its challenges.

Logistic regression is a simple yet effective model, often used for feature selection in microbiome studies. While typically outperformed by more complex models, it remains useful due to its interpretability, especially when identifying potential cancer biomarkers. Regularization methods like LASSO and Ridge help reduce overfitting, making it an efficient choice for small datasets (87) (**Figure 3**).

## 10 Microorganisms and cancer: drivers or companions

The intricate relationship between microorganisms and cancer has been widely studied, but it remains a topic of ongoing debate whether microbes function as drivers (actively contributing to the initiation and progression of cancer) or as companions (simply coexist with tumors without directly causing them). To resolve this ambiguity, researchers must investigate into the biological mechanisms connecting microbes to cancer, distinguish correlation from causation, analyze experimental findings, and address the limitations in current studies. Microorganisms influence cancer development in several ways, predominantly through mechanisms like chronic inflammation, DNA damage, metabolic disturbances, and immune modulation (88). Persistent infections by certain microbes often provoke inflammatory responses, releasing ROS and pro-inflammatory cytokines that can cause DNA mutations and foster tumor growth. For instance, *Helicobacter pylori* plays a role in gastric cancer by inducing chronic inflammation, while *Fusobacterium nucleatum* has been linked to colorectal cancer through immune modulation and enhanced tumor cell survival (89). Additionally, some bacteria, such as *Escherichia coli* strains carrying the pks pathogenicity island, produce genotoxins like colibactin, which generates DNA double-strand breaks, leading to genomic instability. Beyond direct genetic damage, microbial metabolites reshape the tumor microenvironment in diverse ways. While certain bacterial byproducts, such as SCFAs, may exhibit protective effects against cancer, others can stimulate tumor proliferation. A case in point is *Fusobacterium nucleatum*, which influences the metabolic dynamics of CRC by increasing glucose uptake in tumor cells, thereby accelerating their growth (90).

A significant challenge in microbiome-oncology research is differentiating between correlation and action, despite evident biological associations. Many microbes linked to cancer may not initiate tumor formation but rather opportunistic colonizers in the tumor microenvironment. To determine their roles, it is critical to compare microbial presence in early versus advanced tumor stages. Microorganisms consistently found in pre-cancerous lesions prior to tumor development are more likely to be drivers of cancer. Conversely, those primarily detected in late-stage tumors are more likely opportunistic participants. Tumors often create unique conditions such as hypoxia and immune suppression that encourage microbial proliferation, further complicating efforts to determine whether microbes cause cancer or simply adapt to tumor-associated conditions. Longitudinal studies that follow individuals over time are instrumental in unraveling this relationship. For instance, research showing that eradication of *H. pylori* lowers gastric cancer risk provides robust evidence of its role as a driver. However, for many other microbes, such long-term data is lacking (91).

**Figure 4:**
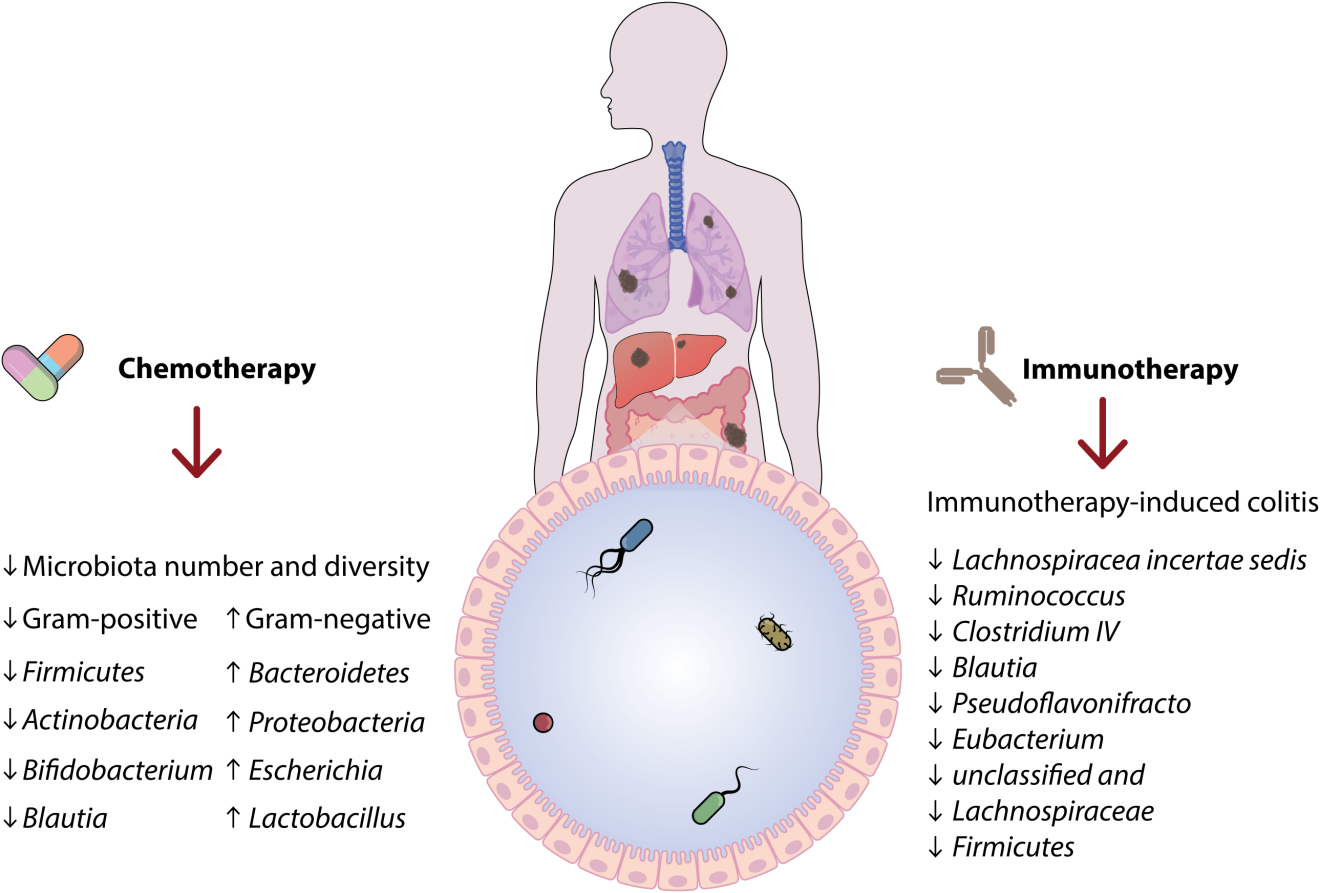
A schematic representation illustrates the two-way interaction between the microbiota and the host immune system. Microbial metabolites like short-chain fatty acids, indoles, and bile acids affect immune responses through pathways including AHR and G-protein coupled receptors. These signals influence immune cell populations, such as regulatory T cells and Th17 cells, playing a role in maintaining immune balance, protecting against chronic inflammation, and potentially impacting cancer development.

Experimental models offer additional insights into the microbiome’s role in cancer. Germ-free mouse models, which are without of microorganisms, develop fewer spontaneous tumors, indicating microbes may play a part in carcinogenesis. Introducing specific cancer-associated bacteria into these models often accelerates tumor growth. For example, introducing *Fusobacterium nucleatum* into mice predisposed to colorectal cancer significantly enhances tumor progression, supporting its role as a driver. Other experimental interventions, such as antibiotic treatments or microbiome transplants, provide further evidence. When antibiotics targeting specific microbes reduce tumor burden, it suggests those microbes have a direct role in cancer progression. Similarly, transplanting a cancer-associated microbiome into germ-free mice and observing increased tumor incidence supports microbial involvement in cancer initiation. Human cohort studies also contribute valuable evidence. Epidemiological links between *H. pylori* and gastric cancer and between *F. nucleatum* and colorectal cancer progression underscore their potential roles as cancer drivers. However, practical and ethical limitations restrict the extent of direct experimental work possible in human subjects (88) (**Figure 4**).

## 11 Conclusion

In conclusion, a deeper understanding of the host–microbiota immune axis lays the groundwork for innovative, personalized, and microbiota-targeted therapies that may transform human health management. Using the potential to revolutionize precision medicine, the integration of microbiome research into oncology has created new opportunities for cancer diagnoses and treatment. The host’s and microbiota dynamic interactions highlight how important they are to the development, progression, and response to cancer therapy. In contrast dietary modification and FMT have become cutting-edge strategies to improve therapeutic efficacy and reduce side effects, microbial biomarkers, such as *F. nucleatum* and *B. fragilis*, demonstrate promising in early cancer detection, especially in colorectal and breast cancers. Significant challenges remain in spite of these developments, such as the inherent variability of microbiota composition between individuals and populations, the lack of standardized procedures, and the requirement for complete verification of biomarkers obtained from microbiota. There is potential for increasing diagnosis precision and prediction treatment results through the creation of integrated diagnostic frameworks that integrate microbiome data with conventional techniques like liquid biopsies and advanced machine learning algorithms. Future research should focus on addressing these challenges by establishing standardized protocols, expanding diverse patient cohorts, and exploring the roles of underrepresented microbial domains, such as viruses and fungi, in cancer biology. Deciphering the intricacies of the interaction between microbiota and cancer will help the field reach its full potential and eventually result in lower-invasive, more individualized, and more successful cancer care approaches.
